# A Manual of Dermatology

**Published:** 1885-07

**Authors:** 


A Manual of Dermatology. *By* A. R. Robinson, M. B., L. R. C P. *and* S. Edin., *Professor of Dermatology at the New York Polyclinic, etc., etc. 8vo, cloth, pp. 647. New York:* Bermingham & Company. 1884.

The author of this new and interesting treatise on diseases of the skin, announces in his preface that this is the precursor of a volume devoted to the same subject, that is to be larger, more pretentious, and more original in character. An examination of the pages before us justifies the opinion that if the succeeding work realizes the promise that is here given, it will assuredly command the success it deserves.

Dr. Robinson has long been known to the profession of this country by his valuable contributions to dermatological subjects made from time to time in the columns of the medical press. Almost without exception these papers have been characterized by a merit due to original and laborious pathological research. As a consequence, few of the later contributions to Dermatological literature of any pretension to a systematic exposition of the entire field, can be compared with the volume before us. In merely glancing over its pages, one is struck with not only the number of illustrations, most of them drawn from microscopical preparations made by the author's hand, but also by the number of subjects to which he has by his own writings made the latest and in many senses the largest contributions. Thus, for example, the brilliant discovery, quite his own, of the existence of both afferent and efferent nerve fibres in the tactile corpuscles, and of the double axis-cylinders in the Pacinian bodies; his well-known establishment of the real relation of several of the derangements of the sweat-secretion to the anatomical seat of the disorder; his clear demonstration of the difference between lichen planus and lichen ruber, an achievement which alone would entitle him to position as a pathologist of high rank,—these results of his untiring industry serve to embellish his pages with a lustre which only too few modern authors can hope to enjoy.

Dr. Robinson, as is well known, is no narrow specialist, but a physician in large general practice, and his pages abound with proofs that his field of labor is not bounded by imaginary lines. His style is terse and effective, his descriptions clear, his suggestions always practical and unbiased by prejudices. His chapter on syphillis is particularly good; and we are pleased to note here that in describing the earliest exanthem of that disease he uses the adjective "macular" instead of "erythematous," in naming the syphiloderma, thus enlisting in the effort to dismiss the abandoned phrase to the old-fashioned category of "syphilitic psoriasis," "syphilitic roseola," and "syphilitic lupus,"

The classification of diseases adopted in the present work is that of Hebra, slightly modified. Of that adopted by the American Dermatological association, the author remarks that it "was decided by balloting, and never should have seen the light." There are many who will not agree with him in this rather sweeping statement. The classification of the American Dermatological Association was modified at its last meeting at West Point, and in the balloting by which position was assigned to each disease, Dr. Robinson took an active part. Like his own, that classification is based on Hebra's system. There has been no classification ever published in this country which can be compared in its educational influence with that of the American Dermatological Association. Hundreds and thousands of young American physicians have acquired, through its aid, a fund of information respecting at least the rudiments of cutaneous medicine. It is not a perfect classification; indeed, none can be framed for years to come which will even approximate such a standard of perfection. At this hour, when a new bacillus is springing into the view of almost every explorer of the pathological field, it is possible to anticipate the day when the present narrow limits of the class in which are found the parasitic disorders may be enlarged till the new class VIII, surpasses in dimensions all of its seven fellows together. But, failing the precise knowledge which such an issue implies and demands, we are sure that the classification which Dr. Robinson thinks should not have seen the light, is still the best working system for the profession at large that it at present possesses.

The work before us is an admirable text-book, and, judged by all standards, can be safely recommended to the practitioner as a valuable aid in the study of diseases of the skin.

